# The Role of Compassion and Mindfulness in Building Parental Resilience When Caring for Children With Chronic Conditions: A Conceptual Model

**DOI:** 10.3389/fpsyg.2019.01602

**Published:** 2019-08-05

**Authors:** Tara M. Cousineau, Lorraine M. Hobbs, Kimberly C. Arthur

**Affiliations:** ^1^Counseling and Mental Health Services, Harvard University, Cambridge, MA, United States; ^2^Center for Mindfulness and Compassion, Cambridge Health Alliance, Cambridge, MA, United States; ^3^Youth, Family and Educational Programs, UCSD Center for Mindfulness, San Diego, CA, United States; ^4^Center for Child Health, Behavior and Development, Seattle Children’s Research Institute, Seattle, WA, United States

**Keywords:** parent, compassion, mindfulness, children with illness/disability, conceptual model, self-compassion, family resilience

## Abstract

Compassion- and mindfulness-based interventions (CMBIs) and therapies hold promise to support parent resilience by enabling adaptive stress appraisal and coping, mindful parenting, and perhaps crucially, self-compassion. These contemplative modalities have recently been expanded to parents of children with chronic illness, building on successful applications for adults facing stress, chronic pain, or mental illness, and for healthcare professionals in response to caregiver burnout resulting from their work. The design and adaptation of interventions and therapies require a conceptual model of parent resilience in the context of childhood chronic illness that integrates mindfulness and compassion. The objective of this paper is to propose and describe such a model. First, we review the need for parent support interventions for this population. Second, we introduce a Model of Compassion, Mindfulness, and Resilience in Parental Caregiving. We highlight the mindful parenting approaches, guiding theories for adaptive coping, and family resilience frameworks that informed our model. Third, we describe a case of a parent to illustrate a practical application model. Finally, we outline future directions for intervention development and research to examine the impact of CMBIs on parent resilience.

## Introduction

A common sentiment is that parenting can be the greatest gift and the greatest burden. This may be especially apt for parents or other caregivers (hereafter described as parents) who are faced with the challenges of caring for a child with a chronic condition, a situation that most families are not likely to anticipate (Myers et al., 2009). Most parents can respond appropriately or skillfully to a short-term childhood health challenge. Vaccinations, chicken pox, stitches, or a broken arm are common experiences and most children recover quickly. With a childhood illness or disability, the picture can be drastically different. Significant parent involvement is often required in daily health-related monitoring or tasks when caring for a child with a chronic condition. In addition to this unexpected burden, parents may face worries about their child’s health and well-being, the loss of certain hopes or dreams for their child’s present and future life, and social isolation because their daily life and experiences are markedly different from other families.

In this context, compassion- and mindfulness-based interventions (CMBIs) hold promise in augmenting parent resilience, which is defined by Rolland and Walsh “as the ability to withstand and rebound from disruptive life challenges, becoming strengthened and more resourceful. Not simply general strengths, resilience involves the dynamic processes that foster positive adaptation in the context of significant adversity” (Rolland and Walsh, 2006). In their analysis of resilience in families of children with chronic illness, they argue that resilience is not just “bouncing back” as popularly defined, but importantly that it “involves struggling, effectively working through and learning from adversity, and integrating the experience into the fabric of individual and shared lives.” Similar to post-traumatic growth, which is defined as a person’s experiences of positive life changes of a traumatic event (Calhoun and Tedeschi, 2006), parents may also experience positive outcomes alongside the suffering and stress of caregiving (Hungerbuehler et al., 2011; Picoraro et al., 2014; Khu et al., 2019). As we will explore in our model, both mindfulness and compassion have significant potential to support this process of working through adversity and finding ways to develop inner resources to cultivate acceptance, find meaning in the context of complex parenting challenges, and respond to the child and oneself with kindness in the face of persistent stressors associated with children’s chronic conditions.

### The Parenting Burden Associated With Childhood Illness

While chronic conditions have been defined and categorized in many ways in the medical literature, there is a common denominator of parental stress and burden across chronic conditions. For the purpose of conceptualizing the role of mindfulness and compassion in building the resilience of parents of children with chronic conditions, we therefore use the broad definition from the National Survey of Children with Special Health Care Needs in the United States (U.S.), which encompasses any condition that has lasted or is expected to last for at least 12 months (Bethell et al., 2015), including mental and behavioral health conditions (e.g., autism or depression), medical conditions (e.g., sickle cell disease), and physical or intellectual disabilities (e.g., cerebral palsy or Down syndrome). An estimated 19.8% of U.S. children have a chronic condition according to national surveys using this definition (Bethell et al., 2014). An estimated 14% of U.S. children have an emotional, mental, or behavioral condition; this prevalence increases to 17.6% in families living in severe poverty, indicating an important disparity (Bethell et al., 2014, 2016). Globally, a meta-analysis of data from 27 countries indicated a pooled estimate of 13.4% of children and adolescents with any mental disorder (Polanczyk et al., 2015). According to a systematic review, one in four children is estimated to experience a chronic pain episode lasting 3 months or more (King et al., 2011).

The association between child chronic conditions and high parent stress is well documented. In a meta-analysis comparing caregivers of children with chronic illness to other caregivers, those who had a child with a chronic illness (asthma, cancer, cystic fibrosis, diabetes, epilepsy, juvenile rheumatoid arthritis and sickle cell disease) reported significantly greater parenting stress, which was associated with greater parent responsibility for managing treatment and poorer psychological adjustment in both caregivers and children (Cousino and Hazen, 2013). Nationally representative studies from multiple countries have documented poor mental and physical health among parents of children with chronic health conditions, activity limitations, and disabilities (Emerson and Llewellyn, 2008; Brehaut et al., 2009; Witt et al., 2009; Yamaoka et al., 2016). Research has also documented lower health-related quality of life (HRQOL), significant stress, post-traumatic stress symptoms, and fears related to a child’s survival (Landolt et al., 2005; Rodriguez et al., 2012; McGrath-Morrow et al., 2013; Guyard et al., 2017).

### Link Between Parent and Child Well-Being

The health and well-being of both parents and their children can be deeply affected by the challenges posed by chronic illness or disability, especially when families cannot engage in normative activities. Some evidence suggests that child health is associated with the physical and mental health of their parents. For example, studies among children with specific conditions have documented an association between poor caregiver mental health and child health symptoms or quality of life for children with asthma (Martinez et al., 2009; Verkleij et al., 2015), type 1 diabetes (Carcone et al., 2012) and cerebral palsy (Turkoglu et al., 2016). Although directionality of the relationship is unclear in such studies, there is some evidence of a bidirectional relationship between mother and child health. For example, a nationally representative U.S. study found direct effects of child activity limitations on maternal activity limitations 2 years later; the inverse was also true (Garbarski, 2014).

Moreover, in the field of behavioral epigenetics, there is emerging evidence that exposure to chronic stress results in enduring physiological sequelae across generations. Studies suggest that parental stress and their history of childhood adversity influence DNA myelination in their children (Berens et al., 2017; Barker et al., 2018). In addition, exposure to non-supportive parenting (high conflict, low warmth and emotion support) at age 17 is predictive of diminished telomere length 5 years later (Brody et al., 2015). Although larger studies are needed to confirm these findings about telomere activity, it is possible that the exposure of children with chronic conditions to their parents’ stress could translate into an impact on children’s health and longevity. No matter the age, an individual’s health and resilience are influenced by both modifiable and unmodifiable factors, including genetic factors, persistence of stressors, learned patterns of coping and appraisal, and access to support (Schneiderman et al., 2005). These findings point to the critical need for interventions to support not just children but the whole family. Interventions designed for parents could therefore impact not only the parent’s mental health and well-being but also child outcomes.

### The Need for Compassion- and Mindfulness-Based Interventions

Parents of a child with a chronic condition may face the challenge of needing to call upon their own compassion to respond to their child’s needs while their own emotional reserves are depleted. Parents may face significant worries about their child’s well-being, suffer when they see their child in distress or struggling, and feel taxed by the constant demands of caregiving. The need to support these parents is well recognized and a variety of interventions addressing modifiable factors have been studied. A Cochrane review of psychological interventions for parents of children and adolescents with chronic illness found that cognitive behavioral therapy (CBT) for parents can improve the child’s symptom reporting for painful conditions; and CBT and problem-solving therapy can improve parent mental health (Eccleston et al., 2015). A meta-analysis on coping support interventions during hospitalizations found reductions in parent anxiety and stress but not depression (Doupnik et al., 2017). Generally, interventions for parents of children with developmental disabilities focus on improving child behavior or teaching positive parenting with the primary outcome of improving child behavior and secondary outcomes of improving parental adjustment, parenting satisfaction and efficacy, and parental relationship (Tellegen and Sanders, 2013; Skotarczak and Lee, 2015). In a similar vein, many interventions aim to increase parents’ medical knowledge or skills related to *treatment adherence* in efforts to improve child outcomes rather than focusing on *parent adjustment*, coping styles, or parenting behaviors (Crandell et al., 2018). However, none of these reviews included compassion- and mindfulness-based interventions (CMBIs) and to our knowledge only a handful of CMBIs have been tested with parents of children with chronic conditions.

Whereas mindfulness interventions are increasingly offered in various settings, compassion-oriented interventions are relatively new and emphasize compassion in relation to the world and oneself in the face of struggle and suffering. Compassion-oriented interventions include Acceptance and Commitment Theory (ACT), Compassion Cultivation Training (CCT), Cognitively-based Compassion Training (CBCT), Mindfulness-based Compassionate Living (MBCL), and Mindful-Self Compassion (MSC), among others (Hayes et al., 2006; Pace et al., 2009, 2013; Jazaieri et al., 2013, 2017; Neff and Germer, 2013; Schuling et al., 2016). Our approach emphasizes relational compassion and self-compassion as we believe that cultivation of safety, connection, and caring is essential in any CMBI or therapy created to support parents when caring for a child with a chronic condition. Compassion for others is typically defined as a feeling of concern for the suffering of another person, coupled with the desire to alleviate that suffering (Goetz et al., 2010). Compassion for oneself involves directing caring and kindness to one’s own distress. According to Neff, self-compassion has three components: mindfulness (being aware of one’s painful experiences in a balanced way), self-kindness (being caring toward oneself), and common humanity (recognizing and understanding that pain and suffering are universal and part of the shared human experience) (Neff, 2003). Compassion interventions also aim to reduce social isolation by increasing a capacity for connection, which is much needed considering the loneliness reported by this parent population (Coke et al., 2013; Neff and Faso, 2014). Perceived social support, an aspect of compassionate behavior, is a potent buffer for stress on health outcomes (Reblin and Uchino, 2008; Seppala et al., 2013; Del-Pino-Casado et al., 2018). While there is relatively little research on the impact of compassion training for parents or other informal caregivers of children and adolescents, the application of these interventions for parents is worthy of study because they appear to reduce burnout in formal caregivers (Jazaieri et al., 2013; Boellinghaus et al., 2014; Raab, 2014; Scarlet et al., 2017).

### A New Model of Compassion, Mindfulness, and Resilience in Parental Caregiving

**Figure 1:**
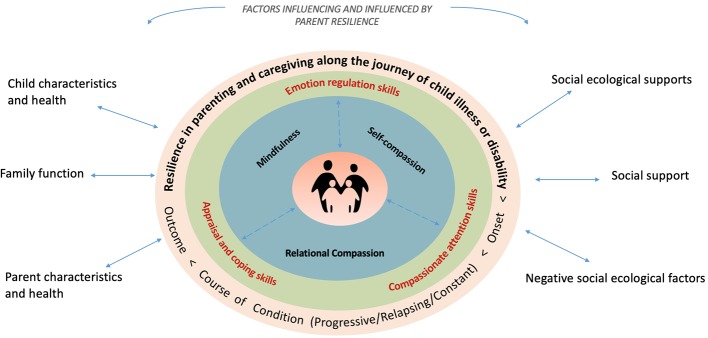
Model of Compassion, Mindfulness and Resilience in Parental Caregiving. The model places the parents in the center, as the source of their own well-being. The blue ring shows the three core areas of skill development in CMBIs. Developing these skills can enable the parent to cultivate three capacities in the green ring – appraisal and coping, emotion regulation, and compassionate attention – which are essential when parenting a child with a chronic condition. There is a bidirectional relationship between these inner resources and the trajectory of a chronic condition (orange ring) because the parent’s resilience can be challenged or further developed in response to changing circumstances surrounding the child’s health and care needs and other contextual factors (outside the rings).

In this context, we propose a holistic Model of Compassion, Mindfulness, and Resilience in Parental Caregiving with the goal of informing how clinicians can support this unique population of parents and how interventions can be developed and evaluated (Figure 1). Our proposed model demonstrates the potential for mindfulness and compassion to foster awareness of the human responses to caring and suffering, acceptance of parental limitations, cultivation of self-care and meaningful experiences of connection with others. CMBIs can thereby enable parents to harness their inner resources for their continued resilience and growth, which is foundational for family well-being in the context of managing childhood chronic conditions. Specifically, CMBIs could enable parents to adopt skillful means and better sustain themselves during the arduous and ongoing tasks of caregiving, engage in mindful parenting, more effectively respond to stressors, and confidently advocate for services.

## Compassion and Mindfulness in Parenting

CMBIs focus on three areas of skill development: mindfulness, relational compassion, and self-compassion. These skills, in turn, support the development of capacities that are necessary for parents facing long-term caregiving challenges with a significant impact on daily life.

### Mindfulness Skills

Our model draws on Kabat-Zinn’s definition of mindfulness as “paying attention in a particular way: on purpose, in the present moment, and nonjudgmentally” (Kabat-Zinn, 1994). Among CMBIs, the study of mindfulness-based interventions has received wide attention especially in helping people manage chronic health conditions and mental distress, such as anxiety and depression (Williams and Kabat-Zinn, 2013). There is a robust evidence base for the feasibility and efficacy of mindfulness-based interventions in improving well-being in adult populations (Fjorback et al., 2011; Hilton et al., 2017; Blanck et al., 2018), as well as children and adolescents (Black et al., 2009; Khoury et al., 2013; Black, 2015; Zoogman et al., 2015; Chi et al., 2018). Twenty years ago, the concept of *mindful parenting* was introduced by Jon and Myla Kabat-Zinn as an alternative to traditional discipline-oriented methods by focusing on the quality of a parent’s presence in the parent-child dyad (Kabat-Zinn and Kabat-Zinn, 1998). Mindful parenting interventions typically focus on cultivating mindfulness and attunement with the parent’s inner experience while interacting with child and feeling the full range of emotions related to parenting. As such, mindful parenting involves cultivating nonjudgmental awareness of the unfolding of internal and external experiences in daily life; practicing emotion regulation skills; learning about adaptive responses to distress; and developing a self-compassionate attitude toward one’s fallibility, limitations, and suffering (Duncan et al., 2009; Bögels et al., 2010). According to Bögels and colleagues, the hypotheses of mindful parenting interventions are that mindfulness training could reduce parental stress, emotional reactivity, parental preoccupation with negative interactions with child, and maladaptive parental dynamics, and improve self-care, parenting skills, attunement to child, and marital function (Bögels et al., 2010). Development of these skills may foster family resilience when managing the daily tasks involved in caring for children with a chronic condition, facing worries about uncertainty in a child’s prognosis, responding to challenging behaviors, navigating competing demands of siblings and family members, and facing stigma and social isolation.

### Relational Compassion Skills

In our model, relational compassion involves responding with kindness and a desire to relieve the suffering of the child, other family members, and those in the community surrounding the family. In the context of parenting, relational compassion may be expressed as a parent’s skillful responses to their child, especially toward experiences that evoke distress, empathy fatigue, shame, and self-criticism when facing daily caregiving challenges. Practicing compassion-focused skills may require simultaneously drawing on one’s ability to be present for the child while responding to the child’s needs or suffering, which can be difficult to do in the moment. Compassion skills may include a recognition of somatic experiences, a focus on breath and calming techniques, and calling on variety of compassion visualizations or meditative practices, such as loving-kindness meditation or imaging a taking away a child’s pain or suffering and offering warmth and joy (Jazaieri et al., 2013). Two mindful parenting models highlight the parallel processes of the parent’s attention to their own experience through mindfulness and the parents’ attention to their child through relational compassion skills. Duncan et al. introduced a framework for mindful parenting with five relational dimensions that has greatly influenced the expansion of mindful parenting into family approaches (Duncan et al., 2009). These dimensions include listening with full attention; nonjudgmental acceptance of self and child; emotional awareness of self and child; self-regulation in the parenting relationship; and compassion for self and child. In a randomized controlled effectiveness study examining the efficacy of an evidenced-based program modified to incorporate mindful parenting compared to the original evidence-based parenting program, participants in the mindful parenting arm had outcomes that were comparable to the original evidence-based program in the domains of interpersonal mindfulness in parenting, parent-youth relationship quality, and parental well-being (Coatsworth et al., 2015). There was a stronger effect for fathers in the mindful parenting arm with respect to interpersonal mindfulness in parenting and relationship quality. In addition, the results from a 1-year follow-up showed that the mindfulness-enhanced program might have greater sustainability of intervention effects on mothers’ ability to monitor their youth. These findings suggest that parents can learn new mindful parenting skills, and potentially over time, enhance natural abilities of awareness, emotion regulation, and dispositional mindfulness in relationship to their children.

### Self-Compassion Skills

In our model, we conceptualize self-compassion as turning toward suffering with an attitude of kindness, compassion, and acceptance, in the same way one might direct care and tenderness toward a loved one or a friend in need, as described by Neff (Neff, 2003). Self-compassion practices also foster an understanding that humans are not alone in their suffering, potentially reducing isolation (Neff, 2003). MSC and Compassionate Mind Training (CMT) are group-based interventions to help people become more self-compassionate (Gilbert and Proctor, 2006; Neff and Germer, 2013). Self-compassion may also be an effective coping strategy to life’s stressors (Allen and Leary, 2010) and has been linked to increased well-being and adaptive coping (MacBeth and Gumley, 2012; Friis et al., 2016; Homan and Sirois, 2017). In the context of family challenges, a parent can form compassionate responses to the caregiving burden as it arises within the self, whether the suffering may be in response to one’s child, within oneself, or in witnessing other families experiencing similar challenges. Parents of children with autism who have greater self-compassion reported greater life satisfaction and hope and less depression and parental stress (Neff and Faso, 2014). An association has also been found between greater self-compassion and lower levels of stress and depression among parents of adults with intellectual or developmental disability (Robinson et al., 2018). CMBIs may increase acceptance of experiences of guilt, resentment, and fatigue related to prolonged caregiving, particularly when such interventions include a focus on self-compassion and normalize caregiving challenges through the sharing of experiences in group interventions.

In summary, mindfulness, relational compassion, and self-compassion represent the core skillsets of the conceptual model and are promising areas for intervention development and evaluation. Although CMBI studies with parents of children with chronic conditions are just emerging and use a variety of approaches, there are consistent positive outcomes among parents of children with autism, developmental delay, attention deficit hyperactivity disorder (ADHD), mental health diagnoses, chronic pain, inflammatory bowel disease, and other chronic conditions (Minor et al., 2006; Benn et al., 2012; van der Oord et al., 2012; Bazzano et al., 2013; Bogels et al., 2014; Dykens et al., 2014; Neece, 2014; de Bruin et al., 2015; Meppelink et al., 2016; Ridderinkhof et al., 2018; Ruskin et al., 2018). Well-being outcomes for parents include greater self-compassion, mindfulness, psychological well-being, psychological flexibility, physical health, life satisfaction, or competence in parenting, and lower parenting stress, overall stress, anxiety, depression, or mood disturbance. These skills and qualities, in turn, are foundational for the dynamic processes involved in developing capacities and building resilience when confronting challenges.

## Parent Capacities Cultivated Through Participation in CMBIs

As the three core skillsets of CMBIs, i.e., mindfulness, relational compassion, and self-compassion, are learned and practiced, parents’ adaptive responses to their child’s needs may influence subtle shifts in internal experiences. These incremental changes over time can lead to better coping, accrual of inner strengths, and enduring beneficial attributes that promote resilience (Hanson, 2013). A primary question raised in the family resilience literature, however, is why some parents cope well with the challenges of caregiving for a child with a chronic condition while others struggle (Rolland and Walsh, 2006; Rosenberg et al., 2013). Even the most well-intentioned or well-resourced parents grapple with prioritizing self-care under demanding circumstances. As illustrated by our model, participation in CMBIs could support the growth of three inner capacities – stress appraisal and coping, emotion regulation, and empathy and compassion – that are essential when parenting a child with a chronic condition.

### Stress Appraisal and Coping

A rich body of literature was spawned with Lazarus’ and Folkman’s classic transactional theory of stress and coping in the face of illness (Lazarus and Folkman, 1984). The theory describes how one responds to, or evaluates, a situation as either benign, beneficial, or stressful (primary appraisals), which is followed by cognitive and emotional processes that influence coping behaviors (secondary appraisals). How a person makes meaning of stress is imbued by one’s personal belief systems, past experiences, and fundamental worldview (Park and Folkman, 1997). Guided by this approach, Thompson et al. developed a transactional model of stress in pediatric illness (Thompson et al., 1992, 1998). Because illness stressors can vary significantly across disease and conditions (e.g., child’s age at symptom onset, type and severity of illness, treatment regimens and life expectancy), this pediatric care model addresses the *adjustment* to illness as a potential stressor (vs. the illness itself). This illness adjustment requires the entire family system to adapt and may involve parameters that are illness-specific or common parameters across illness (Thompson et al., 1998). These adaptations include cognitive processes of stress appraisal, health locus of control, self-efficacy, and self-esteem among others, which could be outcomes of CMBIs but to our knowledge have not yet been explored among parents and caregivers of children with chronic conditions.

The study of self-compassion and mindfulness as coping strategies is a recent line of inquiry (Allen and Leary, 2010; Tharaldsen and Bru, 2012) that aligns with coping appraisal conceptualizations. Consider that Folkman and Greer posited that meaning-making coping “helps the person relinquish untenable goals and formulate new ones, make sense of what is happening, and appraise benefit where possible. This type of coping also generates positive affect, which provides a psychological ‘time out’ from the distress and motivates further coping. An important feature of this positive affect is that it can co-occur with negative affect, perhaps not at the very same moment, but certainly close in time (Folkman and Greer, 2000).” The skill of holding both negative and positive emotions and turning toward suffering with compassion are basic elements in compassion-focused therapeutic approaches (Gilbert, 2009). There is evidence that people high in self-compassion are more likely to use positive cognitive restructuring as a coping skill compared to people low in self-compassion (Leary et al., 2007). Self-compassionate people tend to view stressful situations in a more positive light and are less likely to judge or criticize themselves. Similarly, self-compassion in medical patients was positively associated with instrumental coping, active coping, planning, positive reframing, and acceptance, and negatively associated with denial, behavioral disengagement, and self-blame (Sirois et al., 2015). Further integration of the coping literature with self-compassion and mindfulness models is needed, along with research to explore the possible impact of CMBIs on a parent’s cognitive appraisals, coping skills, and post-traumatic growth.

### Emotion Regulation

In the context of the ongoing challenges faced by parents of children with chronic conditions, emotion regulation is an essential capacity. Emotion regulation is a complex process by which an individual modulates a range of human emotions, consciously and unconsciously. It refers to the processes by which individuals influence which emotions they have, when they have them, and how they experience and express these emotions (Gross, 1998). Harnett and Dawe point out the significance of emotion regulation in their review of 24 mindfulness skills programs for children and families: “The extent to which the parent’s capacity to be emotionally available and ability to consistently implement parenting practices based on fair and reasonable values and expectations is directly influenced by parent’s emotion regulatory capacities” (Harnett and Dawe, 2012). Understandably, a child’s illness or disability may be experienced as traumatic by parents, challenging their sense of efficacy, fairness, identity, and beliefs about the world. Parents can feel overwhelmed when faced with the daily tasks of caregiving, responding to their child’s behavior and needs, and the feeling that there is no end in sight (Robinson et al., 2018). These circumstances can fatigue the family system and result in cycles of emotional distress, anxiety, and depression, leaving family members at risk for burnout (Hamlyn-Wright et al., 2007). Moreover, cultivation and regulation of positive emotions is critical for a parent population facing chronic stress. Tugade and Fredrickson proposed that activation of positive emotions while coping with challenges can foster resilience (Tugade and Fredrickson, 2007). In particular, “positive emotions can momentarily broaden people’s scopes of thought and allow for flexible attention, which can in turn improve one’s well-being. Over time, and with repeated experiences of positive emotions, this broadened mindset might become habitual” (Tugade and Fredrickson, 2007). For example, there is evidence that practicing loving-kindness over 7 weeks activates positive emotions and promotes a range of personal resources in working adults (e.g., increased mindfulness, purpose in life, social support, life satisfaction, and decreased illness and depressive symptoms) (Fredrickson et al., 2008).

### Compassionate Attention

There is a large body of work exploring the benefits of compassion for personal and collective well-being. We draw from the emerging science on compassion in the care of self, care of other, and ability to receive care from others (Seppala et al., 2013; Ricard, 2015). For our purposes, we refer to compassionate attention as the capacity to sustain caring attitudes and behaviors toward oneself and others in daily life, including caregiving and parenting. Compassionate attention may include being aware and accepting of one’s own needs for nurturing and kindness when experiencing caregiver burnout, seeking respite or support from others, and comforting others in similar situations.

Paul Gilbert’s biopsychosocial paradigm for fostering “compassionate patterns” in the brain also serves as a foundation for our conceptual model (Gilbert, 2009) and nicely integrates the capacity for emotion regulation. Gilbert describes the human responses to stress and threats from an evolutionary lens, where individual behavior and responses to the world emerge from deeply ingrained patterns in the human brain passed on from generation to generation over millennia. This has important implications in parenting psychoeducation and skill building. A person’s perception of threat or safety is driven by neurophysiological mechanisms that operate largely beneath awareness; understanding these processes allows for a broader view of human nature as well as cultivating a different, less judgmental awareness of one’s responses to the stressors of daily life. Gilbert purports that there are three types of emotion regulation states in the brain that are in a continual push and pull dynamic to achieve balance. These three systems include: (1) a threat and protection system that serves as a kind of background surveillance of potential threats and harms, (2) an incentive and resource-seeking system that functions to support positive feelings and motivation to seek out resources and pleasure, (3) and a soothing and contentment system, that serves to restore balance, calm, and caring connection to others, and is associated with compassion and kindness. Through skills that foster awareness of these human processes, individuals can respond to distress with greater skill, and learn to nurture themselves and others. The development of such skillful means is supported by a systematic review indicating that self-compassion is negatively associated with emotion dysregulation and positively associated with adaptive emotion regulation (Inwood and Ferrari, 2018).

### Helping Parents Turn Skills to Strengths

In our model, the parents who are exposed to the core skills of CMBIs can cultivate capacities for coping and appraisal, emotion regulation, and compassionate attention, which may lead to more enduring resilience. Skills for augmenting healthy emotion regulation and soothing of self and others play an important role in the parent-child interaction (Gordon, 2009). For example, breathing skills and open awareness practices may lead to trait mindfulness, and compassion-focused meditations can more readily evoke positive feeling states, such as love, gratitude, or self-kindness. This is especially true in relation to empathy. When parenting a child with a chronic condition, there are many circumstances in daily routines and interactions with the school and healthcare systems when a parent may witness the suffering of their child. In such situations, the parent may experience empathic distress, which occurs when an unpleasant and charged reaction to suffering, distress, or pain witnessed in another causes a cascade of physiological reactions that activate similar neural networks associated with responses to physical pain (Singer et al., 2004). When confronted with a distress trigger, one is more likely to experience negative emotional states, such as aversion, danger disgust, fear, or withdrawal. The distress can lead to empathy fatigue over time when there is little or no respite and coping may diminish. For example, parents of children with chronic pain syndrome often feel upset or helpless watching their children suffer (Palermo and Eccleston, 2009; Noel et al., 2016; Palermo et al., 2016). Parents may also inadvertently reinforce a child’s “passive sick role,” by giving special attention to the child, such as reduced responsibilities at home (i.e., chores) or opting out of normative social activities, which is associated with absenteeism and lower levels of school functioning (Van Slyke and Walker, 2006; Logan et al., 2012). Conversely, empathic concern is a compassionate response to suffering and is associated with positive emotions, such as warmth, caring, and connection. Evidence is emerging for the role of self-compassion in emotion regulation. For example, in a review of five studies, emotion regulation was shown to mediate the relationship between self-compassion and mental health (Inwood and Ferrari, 2018). For parents of children with chronic conditions, self-compassion has the potential to help alleviate persistent feelings of despair, helplessness, and chronic fatigue in caregiving. In daily practice, there is an art and skill to moving between moments of empathic distress to a state of compassion, whether the one suffering is another person or oneself (Singer and Klimecki, 2014).

## Factors Influencing Parent Resilience

While CMBIs provide skills that can be foundational for the development of essential capacities for caregiving and parenting, the dynamic processes of resilience are affected by the context of the family and community as well as the trajectory of the child’s illness or disability. As described by Raina et al., a parent or caregiver’s resilience is influenced by parent characteristics, familial function, child characteristics, social support, and social ecological factors (Raina et al., 2005). To consider the role of CMBIs in building resilience for this population, we examined key integrative family system models that include multiple facets of caregiving burden and consider the potential trajectory of illness or disability, which can vary significantly by diagnosis or condition.

### Family Systems-Illness Model

Rolland and Walsh proposed “an integrative developmental system model to help children and families meet their illness-related challenges” (Rolland and Walsh, 2006). The FSI model uses a resilience framework for pediatric healthcare developed at the Center for Illness in Families at Yale University and the Center for Family Health at the University of Chicago. This model draws on the therapeutic shift in family therapy from addressing deficits to a focus on strengths. Their intention is to support the function of the family as a collaborative team, which is critical for the best psychosocial and/or health outcomes in the context of childhood illness, disability, or loss.

Given that the trajectory of a child’s illness or disability is often uncertain and prolonged, a parent naturally vacillates between present caregiving needs and future considerations. Parents also face some challenges that are constant but may evolve over time as the child ages (e.g., challenging behaviors in the context of developmental delay or the emergence of adolescence) and other challenges that are acute (e.g., a hospitalization). Importantly, the FSI model also takes into account illness-related patterns of the psychosocial demands by using a typology of chronic or life-threatening illness. Depending on the child’s condition, the *onset, course,* and *outcome* of an illness may vary. An advantage in this approach for clinical practice and intervention is finding a “goodness of fit” between the psychosocial demands that a family can experience over the course of the child’s condition and the strengths/vulnerabilities of the family. For example, a child or adolescent may have a gradual or progressive disease (childhood cancer; cystic fibrosis), a constant course condition in which an initial event is followed by a stable course (cerebral palsy; spinal injury; phenylketonuria) or one with a relapsing or episodic course (asthma; migraine; chronic pain). The *outcomes* also vary, and depending on the condition, may lead to prolonged care and disease management, death, or shortening of lifespan. Disability can involve *incapacitation,* and the degree of impairment can vary across a number of domains, including cognition, sensation, movement, stamina, and social stigma. The FSI model delineates three time phases that can help clinicians view the bigger picture: crisis (diagnosis and adjustment), chronic (the long haul), and terminal (pre-terminal, death, mourning, and resolution of loss). Naturally, each phase requires sensitivity to meet the needs of parents and family members, including siblings; support individual coping strategies; and employ age-appropriate strategies to share information with children and meeting the need for autonomy of all members. Similarly, researchers at the University of Australia proposed a model adapted from the Supportive Care Needs Framework for parenting a child with a rare disease and addressed a cohort of parents who are often overlooked (Pelentsov et al., 2015). The majority of rare diseases affect a child’s life from birth. Rare diseases may also impact children and families in unique ways. For example, due to limited information and research about their child’s condition, these parents may find themselves in the unexpected role of becoming an “expert” on the disease who must then inform providers about their child’s needs. In some cases, parents themselves may have the rare disease and associated physical symptoms, creating the double burden of needing to receive care and serve as a caregiver.

### A Multidimensional Model of Caregiving Process and Caregiver Burden

Raina et al. made an important contribution to the literature by focusing on the unique caregiver stress and burden in the pediatric population. Like the FSI model, they also consider multiple factors influencing the psychological and physical health, but with explicit sensitivity to the parental experience of caring for children with chronic illness (Raina et al., 2004, 2005; Klassen et al., 2007). In their view, “the characteristics of the caregiver, the recipient of care, their shared history, and the social, economic and cultural context within which they find themselves combine to create an infinite variety of circumstances from which stress may both originate and be managed.” In their model, stress is conceptualized as the intersection between a caregiver’s external environment and internal states. For example, a child’s condition can greatly challenge the caregiver’s subjective response in the caring role; or alternatively, stress may ensue when the demands of caregiving interfere with a parent’s sense of identity and pursuit of other goals in adulthood. Their model suggests that social ecological context, family context, and child-related factors must be considered when developing and evaluating interventions. Family resilience models offer a lens in addressing the complexity and the contexts in which suffering of caregivers and families can arise, which CMBIs are well suited to address.

## Translating the Model: A Case Example

To highlight the breadth of parental burden and resilience, it is helpful to consider a parent’s experience relative to some of the specific conditions that have been described in the literature on chronic conditions. For example, in a child with an emotional or behavioral health problem, parents may struggle with treatment decisions due to their past experiences or beliefs. A parent may be uncertain about getting treatment due to the belief that the child will overcome the issue without treatment, may believe that a child’s mental health condition is in response to a stressful event in the family, or may harbor guilt about the impact of the parent’s own behavior on the child’s mental health (Mayberry and Heflinger, 2013). In the case of a child with a condition that can have both a physical and intellectual impact like cerebral palsy, parents may need to provide assistance with feeding, help their child with other daily tasks impacted by motor delays, and navigate comorbid behavioral health challenges (Guyard et al., 2017). How might a CMBI informed by our model promote parental resilience? We next describe a composite case based on the authors’ collective experience in facilitating and designing CMBIs (Bluth et al., 2016; Seidman et al., 2019).

### Composite Case: A Mother of a Child With Rare Gastrointestinal Disease

We begin with a composite case^1^ of a mother to help illustrate the model, followed by a discussion of each ring in the model, as depicted in Figure 1.

Sofia came to a compassion and mindfulness program for parents of children with health conditions in a state of complete exhaustion. Her younger son Alex, now 5 years old, required a nasogastric feeding tube for 6 months after birth due to severe gastrointestinal problems, which meant that Sofia could not breastfeed. When he was 8 months old, she and her partner made the difficult decision to have a feeding tube surgically placed in his stomach. After 2 years of insistence from medical professionals that Alex had nothing more than gastroesophageal reflux disease, he was diagnosed with a rare gastrointestinal condition.

By now Sofia is weary of the exhaustive efforts in helping Alex learn to eat in order to wean him from the feeding tube. When sitting with Alex at mealtimes, Sofia finds herself saying to herself, often aloud, “I can’t do this, I give up.” In the hardest moments, she retreats to the bathroom so her family will not see her crying. Since starting kindergarten Alex’s diet and routine have changed. He has lost weight and Sofia feels they have lost ground. The school nurse does not have enough time to create a positive eating experience as recommended by the feeding therapists. Sofia is also worried he will be rejected by his new classmates for being different.

As Sofia engages in learning mindfulness and compassion in the parenting program, she experiences the relief of being in a room with other parents who have a variety of challenges with their children. Sofia learns that they also feel worried, frustrated, guilty, sad, and angry. The group facilitator demonstrates a quality of warmth, calm, and containment. For the first time Sofia does not feel judged, but begins to understand the meaning of common humanity, *we are all in this together.* Building new skills, however, is not easy. During meditation, Sofia finds herself distracted due to feelings of guilt about being away from home at Alex’s mealtime. No matter how hard she tries to believe the kind and supportive words from friends and medical professionals, she cannot help but think that she must be doing something wrong when she tries to feed him.

After starting the program, she is increasingly aware of her inner dialogue at home. She notices that her thought patterns are full of judgment and self-criticism. *This is my fault. I’m a terrible mom. What am I doing wrong? The therapists say I should make meals fun, but I always get so frustrated. If I took better care of myself, I would be more patient. But how am I supposed to find the time?*

Sofia begins to experience a shift as she practices the self-soothing strategy of putting her hand on her stomach whenever she feels tense. She learns that this soothing gesture along with a kind intention toward herself releases important hormones that have a calming effect. A kinder voice arises within her. She acknowledges that while life with Alex is hard, her feelings of fear, anxiety, and overwhelm are natural under the circumstances. Her perspective shifts when in one session, another mother points out that the feeding tube is not so terrible because the tube ensured that her child could obtain the needed calories to grow while experiencing and learning to enjoy new foods. This new perspective eases Sofia’s resentment about the feeding tube and she becomes more hopeful about the future. Her anxiety lessens and she sleeps better. Sofia finds herself able to think more clearly about the best path forward. She advocates for a special aide at school to manage the feeding protocol and requests that Alex receive tube feeds in privacy so other children will not stare. Sofia feels renewed in her commitment to advocate for her son.

Things change at home, too. Sofia becomes more sensitive to Alex’s reactions to food, noticing when he seems to be struggling with food textures. Her new awareness and ability to calm her own fears help her when struggling with Alex. When she notices a negative inner voice, she often remembers self-compassion statements she created in the program, such as, “I’m doing the best that I can.” The family also starts to have meals together instead of feeding Alex separately. Sofia now spends more quality time with her older daughter and even teaches her to how cultivate a “compassionate friend” as a bedtime meditation. After the program ends, Sofia continues to take compassion breaks during the day. In difficult moments, she recalls the faces of the parents and asks herself, “What would they say to me right now?” Sofia’s inner voice of compassion answers with a deep knowing and sense of warmth and caring, “This is so hard right now, but it is going to be OK. I am not alone.”

We now apply the model (Figure 1) to understand this parent case, starting from the center and moving out.

#### The Parent/Caregiver (Center)

In our model, Sofia, the parent, is at the center. As the expert on her own experience in caring for her child, the assumption is that she is seeking reinforcement of her own inner wisdom and motivation for self-care. Resilience is not simply returning to a preexisting level of well-being but rather bouncing forward to greater capacity, strength, and personal growth as parents face the shifting challenges over the course of a child’s condition and childhood. As Sofia builds her skills, she implements new strategies for healing and well-being. Parents of children with chronic conditions are in continual state of worry and vigilance as they “face the formidable challenge of focusing on both the present and the future” of their child (Rolland and Walsh, 2006). Caught on a tightrope of the daily tasks of caregiving and future planning, parents like Sofia are in an ongoing balancing act. These situations are fertile ground for rumination, hopelessness, and social isolation, which could be reduced by exposure to CMBIs (Hilt and Pollak, 2012).

#### Introduction of CMBI Skills (Inner Blue Ring)

Sofia joins a program designed for parents that includes content and practices related to developing skills related to three core areas: mindfulness, relational compassion, and self-compassion. CMBI therapies (e.g., ACT, MBCT, Compassion-Focused Therapy) and group interventions (e.g., mindful parenting, MSC, MBSR, CCT, MBCL), or hybrid parenting interventions serve to help individuals cultivate inner resources and emotionally nourishing states of wellbeing as essential to self-care. As a felt experience or quality of being, mindfulness and compassion are inseparable. We recognize it is largely a matter of emphasis among the CBMI repertoire. The psychologist and meditation teacher Tara Brach describes mindfulness and compassion as two wings of a great bird that enable “coming home to loving presence” (Brach, 2016). Moreover, how a therapist or group facilitator embodies these qualities of awareness – seeing clearly and holding one’s experience with compassion – may be a potent source for parents in acquiring skills (Pollak et al., 2014). Sofia learns not only from the other parents, but from the gentle guidance of the teacher. With practice of CMBI skills over time, “states” can be turned into enduring “traits” fostering post-traumatic growth (Hefferon et al., 2009; Garland et al., 2010; Hanson, 2013).

#### Coping, Emotion Regulation, and Compassionate Attention (Green Ring)

Participation in CMBIs supports the development of essential capacities: adaptive stress appraisal and coping, emotion regulation, and compassionate attention. For example, Sofia learns *emotion regulation skills* related to her breathing and resting her hand on her belly. Mindful awareness skills taught in CMBIs are intended to engage parasympathetic responses, and offer new ways to relate to experiences that are present moment-focused rather than avoidant. In the context of daily or episodic challenges related to a child’s health and caregiving, emotion regulation may be supported by mindfulness skills, such as noticing and accepting difficult thoughts or emotions and practicing self-soothing activities, e.g., getting rest, listening to music, paying attention to the breath, or practicing a brief body scan meditation (Shapiro and White, 2014). Sofia begins to identify her emotions and to take a step back from the intensity of a stressful parenting or caregiving situation, take short breaks (i.e., parent “time-outs”) to relieve stress, and practice present moment awareness during mealtimes with Alex. In time, these practices may also foster dispositional mindfulness in Sofia, and in turn, promote greater clarity, calm, and coping in day-to-day caregiving, enabling her to assess and reassess goals as Alex grows up or the condition changes, and planning for future challenges (e.g., entering the school system and planning for independent living as a young adult).

One of Sofia’s greatest challenges is weaning Alex from his feeding tube. When it comes to a child’s needs, compassion skills may foster warmth and caring in challenging situations (Gilbert, 2009), an openness to understanding the child’s perspective and need for autonomy, greater tolerance for empathic distress, and less parental solicitousness. Parents of children with chronic conditions may benefit from such strategies when facing acute issues, such as challenging behaviors, medical crises or a child’s pain, as well as persistent long-term worries, such as concerns about who would take care of the child if the parent becomes ill, incapacitated, or dies. For parents like Sofia, who struggle with promoting child autonomy and boundary-setting due to disabilities or treatment regimens, greater emotion regulation and psychological flexibility enable them to better respond to conflicts that arise over roles or responsibilities or stay in the present moment without being overcome by a child’s pain or difficulties (McCracken and Gauntlett-Gilbert, 2011).

As Sofia engaged with other parents in an environment of safety, she listened to their stories and learned from them. She began to cultivate adaptive *illness-related appraisals* and cope better. She experienced a shift in mindset when hearing another mother’s perspective about weaning her child off of a feeding tube. This positive appraisal helped Sofia reassess her own beliefs in how she related to the feeding situation. It also shifted her behaviors and attitudes about managing mealtimes, resulting in more pleasant experiences for the entire family. As was suggested by Duncan et al. in their mindful parenting model (which includes the components of *listening with full attention* and *compassion for self and child*) (Duncan et al., 2009), mindfulness may enable parents to use more adaptive appraisal and coping skills in relation to the child, the situation, or themselves. In addition to responding to the immediate caretaking needs of their children, parents may engage in action-oriented coping, such as addressing situations that arise in caregiving or with schools, engaging in advocacy and building community.

As Sofia begins to notice a critical inner voice about her mothering, she is cultivating *compassionate attention.* Parents can apply self-compassion practices in specific situations that may evoke shame or guilt, such as feeling judged by others about their parenting, receiving unsolicited advice, self-blame about not doing enough for their child, or fear of making the wrong treatment decisions. Informal self-compassion practices can include a moment of breathing with the hand over the heart, use of personalized compassion phrases, soothing touch, or compassion breaks (Neff and Germer, 2018). Considering the challenging life circumstances of parents of children with chronic conditions, formal compassion practices, e.g., loving-kindness meditations or compassionate body scan, may be more difficult to apply with time constraints, but experiences with these meditations in a class could provide a learning opportunity that allows for informal use of these practices in difficult moments. Other compassion meditations call on use of imagination. Caring imagery is intended to evoke positive feelings. These visualizations can induce a kinder inner voice and compassionate attributes, such as tenderness and warmth, non-judgment, safety, strength, inner courage and wisdom (Gilbert, 2009, 2010; Neff and Germer, 2018).

#### Illness and Disability Trajectories (Orange Ring)

Wrapped around these capacities is the parent’s journey along the illness trajectory, depicted by the outer orange ring. Sofia is facing the challenge of Alex starting school, which requires him to be more self-sufficient as he grows up and also involves relying on school personnel to be both sensitive and skillful regarding his basic needs. The application of the core skills and cultivation of enduring capacities can benefit parents along the unpredictable journey of the child’s illness or disability as described by the Family Systems-Illness model, e.g., symptom onset, diagnosis, course of illness, and outcome, including incapacitation or loss (Rolland and Walsh, 2006). Strengthening adaptive appraisal and coping, emotion regulation, and compassionate attention may buffer the stress of uncertainty. There may be a virtuous cycle of well-being as a parent becomes more fluent in a mindfulness or compassion skill. The practice of CMBI skills may enhance these three capacities and *vice versa*, in a bidirectional and dynamic manner.

#### Factors Influencing and Influenced by Parent Resilience (White Area)

Sofia feels safe and understood in the parenting group as she learns about other parents’ unique situations and shares in their struggles. The factors listed outside the circle in which a parent is contextually situated with arrows suggest that they impact the parent’s resilience along the journey. As parents practice informal or formal skills offered by CBMIs, they may experience stress reduction and improvements in well-being. They may also experience beneficial changes in intrapersonal and interpersonal dimensions of caring for a child with a chronic condition and interacting with family members and others involved in the child’s care. The application of these skills supports the key adaptive processes in family resilience, including belief systems (making meaning of adversity, positive outlook, transcendence, and spirituality), organizational patterns (flexibility, connectedness, and social and economic resources), and communication/problem-solving (clarity, open emotional expression, and collaborative problem-solving) (Rolland and Walsh, 2006). Notably, social isolation and stigma are frequently experienced by this population of parents, and fear of compassion may also arise (i.e., parents may find practicing self-compassion and receiving compassion difficult). As such, the recognition of common humanity that emerges from engaging with other parents offers an opportunity to both offer and receive compassion and may be a key mechanism supporting resilience by normalizing difficult emotions and recognizing that one is not alone (Neff and Germer, 2013). The importance of fostering beneficial experiences and positive emotions in the experience of caregiving cannot be overstated, and this may be best served in caring communities.

## Future Directions

We have suggested that CMBIs could support the development of foundational skills that enable greater resilience in caregiving and parenting a child with a chronic condition. There is an opportunity to build on the existing interventions for this population and learn from interventions in other populations – particularly compassion-focused interventions – to reach more families in need and improve additional outcomes related to parenting and caregiving in the context of chronic illness or disability. We therefore advocate for (1) development or adaptation of compassion-focused interventions for this population; (2) continued development of tailored interventions in partnership with parents and other caregivers; and (3) research to examine the impact of these interventions on parent and child resilience.

### Development or Adaptation of Compassion-Focused Interventions

Mindful parenting interventions have laid the foundation for further development of interventions to include a stronger focus on self-compassion and relational compassion to foster parent resilience, self-care, and coping skills. Self-compassion practices hold the potential to ease feelings of guilt, shame, or disappointment that may plague parents who are struggling to make meaning of the life-long challenges that are unlike those of other families around them. Relational compassion, through connection with other parents, may help with some of the unique challenges of parenting a child facing medical issues (e.g., pain episodes, medication, or therapy adherence) or developmental or behavioral challenges (e.g., internalizing or externalizing behaviors). Group interventions that include activities promoting a sense of common humanity may be particularly healing, with the shared recognition that other parents facing child health challenges are also struggling to reduce feelings of isolation. In addition, the cultivation of relational compassion could lead to greater understanding of others involved in the child’s life, medical care, or school. This, in turn, may help parents navigate complex relationships within the wider community.

### Development of Tailored Interventions

Considering the social isolation, stigma, and unique medical, developmental, or behavioral challenges faced by parents of children with chronic conditions, they are likely to feel most comfortable in settings with peer parents. In fact, support groups for parents of typically developing children (e.g., for new parents or parents of toddlers) could have the unintended consequence of causing additional feelings of grief and loss if parents facing these unique challenges are exposed to conversations about the typical challenges of childhood. In light of the difficult emotions that come with raising a child whose daily life and activities are impacted by his/her health condition or disability, parents may find great benefit from tailored interventions that address feelings such as grief or loss, resentment, frustration with child behaviors, or anxiety about the future and provide an opportunity for parents to see that other parents are experiencing similar feelings. Much can be learned from tailored interventions that have been tested. Benn et al. and Bazzano et al. described adaptations to Kabat-Zinn’s Mindfulness-Based Stress Reduction (MBSR) curriculum that were specific to this population, integrating concepts such as emotion regulation, forgiveness, kindness, and compassion and incorporating group discussions about stress or concerns related to the child and their future (Benn et al., 2012; Bazzano et al., 2013). These researchers found improvements in self-compassion following the intervention. Of note, Benn et al. included mindfulness training for teachers, recognizing the importance of coordinating parent and teacher interventions to optimize child outcomes (Benn et al., 2012). Considering that many families of children with chronic conditions have frequent interactions with therapists (e.g., occupational or speech therapy), medical providers, and school professionals, interventions that support the mindfulness and compassion of these professionals could prove beneficial personally and professionally, alleviating caregiver burnout and developing their capacity to provide a mindful and compassionate presence to families.

Collaborating with parents and other caregivers in the design of these interventions is essential because their experiences and challenges are very different from parents of children without chronic conditions. For example, Bazzano et al. developed their curriculum using a community-based participatory approach that involved collaborating with parents of individuals with developmental disabilities who participated as equal partners on the project planning committee, guiding the program and research design and implementation (Bazzano et al., 2013). The intervention resulted in a significant reduction in perceived stress and in parental stress and increased mindfulness, self-compassion, and well-being. Dykens et al. trained parents of children with disabilities to deliver MBSR or Positive Adult Development (based on positive psychology) in a randomized controlled trial comparing the efficacy of the two interventions (Dykens et al., 2014). Collaboration with parents and caregivers is also important to ensure the design and evaluation of CMBIs that are culturally relevant for communities of color. To our knowledge, most interventions have been conducted with predominantly Caucasian participants. Most studies with culturally diverse populations did not examine differences in outcomes based on race, ethnicity, culture, or religions, with the exception of a study by Neece et al. (2019) that found that participation in MBSR with simultaneous English-Spanish interpretation resulted in improved mental health for Latino parents of children with developmental delay. Formative research is needed to explore the extent to which mindfulness and compassion practices align with the cultural or spiritual values of communities of color and inform the design of culturally relevant interventions.

Participation of parents and caregivers in the design process could also help address barriers to participation to ensure that CMBIs are both accessible and impactful. If these barriers are not addressed, these interventions could have the unintended consequence of increasing health disparities, with the most privileged families having improved outcomes as a result of participation in CMBIs while the most disadvantaged families continue to experience high levels of stress and poor mental health. First, there are issues associated with poverty and geography. Chronic conditions are more prevalent among children facing poverty and trauma (Bethell et al., 2014), and their families are less likely to have the time and resources needed to participate. In addition, these programs are primarily offered in urban areas and in major medical academic centers, making access difficult for families in rural areas, who may face the greatest isolation and may have more challenges in accessing services. Second, parents of children with chronic conditions face significant challenges in caring for their child and obtaining child care. It may not be possible to attend weekly sessions, participate in a standard 8-week CMBI program, or engage in daily formal meditation practice. Other formats should be explored for feasibility and impact. For example, a feasibility study examining a one-time, 2-hour mindful parenting workshop for parents of an adolescent suffering from chronic pain and inflammatory bowel disease paired with a concurrent mindfulness workshop for adolescents found satisfaction with the program and immediate benefits for aspects of psychological flexibility although no change in mindful awareness (Coakley and Wihak, 2017). Other CMBI innovations include technology-based tools and resources that may offer overburdened or under-resourced parents with compassion and mindfulness skills training, psychoeducation, and a sense of community. For example, a single-arm feasibility pilot of a mobile intervention for parents of children with chronic pain revealed significant decreases in parental solicitous behavior and perceived stress, and a significant increase in mindful parenting (Seidman et al., 2019).

### Research Examining the Impact of CMBIs on Parent and Child Resilience

Research is needed to explore the hypothesized relationships underlying our model and to determine whether participation in CMBIs results in greater resilience for this population. Cross-sectional studies such as path analysis offer one way to examine direct and indirect associations between the constructs in the model. This can be challenging given that interventions include a variety of skills and techniques, and measurement tools have overlapping constructs. However, research could explore questions such as whether parents with high dispositional mindfulness show more adaptive coping and greater emotion regulation in challenging situations or when facing long-term worries about their child, or whether parents with high self-compassion show greater emotion regulation, more adaptive coping, and greater relational compassion in parenting. Research could also examine the relationship with resilience and post-traumatic growth by exploring whether parents with more skills related to emotion regulation, appraisal, and coping, or compassionate attention show greater resilience. In addition to measuring the impact of CMBI participation on parents, research is needed to measure the impact on the child or the siblings.

In summary, this article describes efforts to extend and integrate previous conceptualizations of parent resilience in caregiving through the lens of compassion and mindfulness-based frameworks. Our model for parental resilience suggests that the outcomes for parents, children, and the family system as a whole may be improved by helping parents cultivate compassion and mindful awareness in the context of caregiving. As one mother said after a long hospitalization for her child with a rare heart condition, “We can live our lives in a constant state of worry and fear for an outcome that is beyond our control, or we can choose to live in the present moment so we can take in all that is beautiful in our lives.”

## Author Contributions

TC takes responsibility for the writing and submission, contributing a significant portion of the work. KA is the principal scientist, contributing a significant portion of the review of the burden of pediatric chronic conditions and the future directions. LH is an expert in mindfulness and self-compassion and family systems, and served as advisor and contributed to portions of the writing and review of drafts. All three contributed to the creation of the conceptual model and image.

### Conflict of Interest Statement

The authors declare that the research was conducted in the absence of any commercial or financial relationships that could be construed as a potential conflict of interest.

The reviewer CL declared a past co-authorship with one of the authors LH to the handling editor.

## Footnotes

^1^The composite case is based on the authors’ combined experiences with this population. No original data or descriptions are included in the case study.
